# Supplementary material for: Efficacy of HE4, CA125, Risk of Malignancy Index and Risk of Ovarian Malignancy Index to Detect Ovarian Cancer in Women with Presumed Benign Ovarian Tumours: A Prospective, Multicentre Trial
Source: J Clin Med. 2019 Oct 25;8(11):1784. doi: 10.3390/jcm8111784 (PMC6912210; doi:10.3390/jcm8111784)
Supplement: Supplementary file 1 [file jcm-08-01784-s001.pdf]

SUPPLEMENTAL DATA

Additional Supplemental Data may be found in the online version of this article:

Supplemental Figure 1. ROC curves of serum markers and algorithms (n=218).

Supplemental Table 1. Histological type of presumed benign ovarian tumor.

Supplemental Table 2. Comparison of the specificity of the tumors markers and algorithms.

Supplemental Figure 1. ROC curves of serum markers and algorithms (n=218).

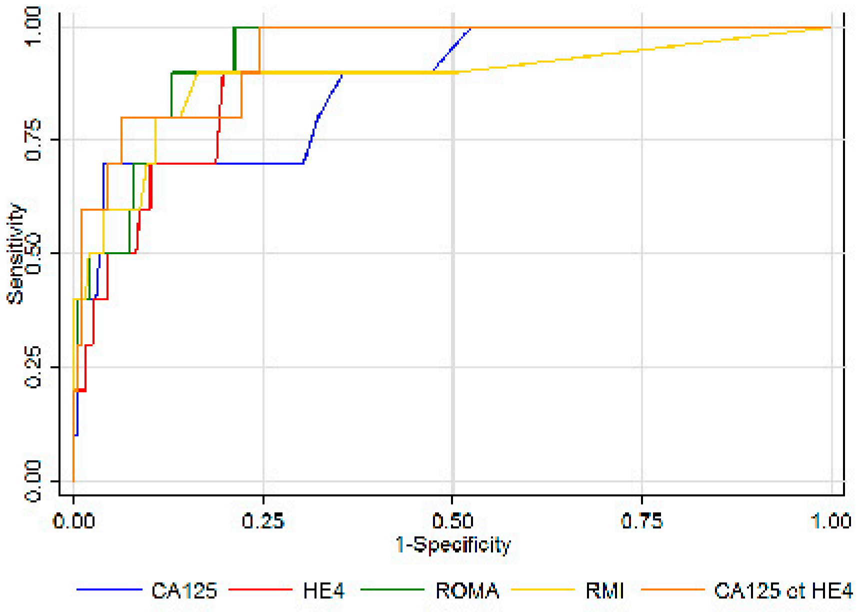

Table S1. Histological type of presumed benign ovarian tumor.

**Histological findings**			**n (%)**
**Benign tumors**			**209 (94.6)**
		Serous cystadenoma	78 (35.3)
		Mature teratoma	44 (19.9)
		Endometriotic cyst	24 (10.9)
		Mucinous cystadenoma	21 (9.5)
		Functional cyst or corpus luteum	17 (7.7)
		Paratubal or inclusion cyst	15 (6.8)
		Ovarian fibroma or fibrothecoma	8 (3.6)
		Other	2 (0.9)
**Malignant tumors**			**12 (5.4)**
	Borderline tumors		10 (4.5)
		Serous tumor	7 (3.2)
		Mucinous tumor	2 (0.9)
		Endometrioid tumor	1 (0.5)
	Malignant tumors		2 (0.9)
		Adenocarcinoma	2 (0.9)

Data are n (%).

Table S2. Comparison of the specificity of the tumours markers and algorithms.

	**Total (n=221)**<br>Specificity (%) [IC 95%]	**Pre-menopausal women (n=141, 63.8%)**<br>Specificity (%) [IC 95%]	**Post-menopausal women (n=80, 36.2%)**<br>Specificity (%) [IC 95%]
CA125	90.4 [85.6-94.1]	87.5 [80.7-92.5]	95.9 [88.5-99.1]
HE4	91.4 [86.7-94.8]	87.5 [80.7-92.5]	98.6 [92.6-100]
CA125 + HE4	99.5 [97.4-100]	100 [97.3-100]	98.6 [92.6-100]
RMI	99.0 [96.6-99.9]	99.3 [95.9-100]	98.6 [92.6-100]
ROMA	83.3 [77.5-88]	75.7 [67.6-82.7]	97.3 [90.5-99.7]
